# Size characterization of plasmonic nanoparticles with dark-field single particle spectrophotometry

**DOI:** 10.1038/s41598-022-21649-8

**Published:** 2022-10-24

**Authors:** Rodrigo Calvo, Andreas Thon, Asis Saad, Antonio Salvador-Matar, Miguel Manso-Silván, Óscar Ahumada, Valerio Pini

**Affiliations:** 1 Mecwins, Roda de Poniente 15, Tres Cantos, 28760 Madrid, Spain; 2 Departamento de Física Aplicada, Universidad Autónoma de Madrid, Campus de Cantoblanco, 28049 Madrid, Spain

**Keywords:** Spectrophotometry, Nanoparticles, Nanophotonics and plasmonics

## Abstract

Plasmonic nanoparticles are widely used in multiple scientific and industrial applications. Although many synthesis methods have been reported in the literature throughout the last decade, controlling the size and shape of large populations still remains as a challenge. As size and shape variations have a strong impact in their plasmonic properties, the need to have metrological techniques to accurately characterize their morphological features is peremptory. We present a new optical method referred as Dark-Field Single Particle Spectrophotometry which is able to measure the individual sizes of thousands of particles with nanometric accuracy in just a couple of minutes. Our method also features an easy sample preparation, a straightforward experimental setup inspired on a customized optical microscope, and a measurement protocol simple enough to be carried out by untrained technicians. As a proof of concept, thousands of spherical nanoparticles of different sizes have been measured, and after a direct comparison with metrological gold standard electron microscopy, a discrepancy of 3% has been attested. Although its feasibility has been demonstrated on spherical nanoparticles, the true strengthness of the method is that it can be generalized also to nanoparticles with arbitrary shapes and geometries, thus representing an advantageous alternative to the gold-standard electron microscopy.

## Introduction

Nowadays, plasmonic nanoparticles are widely used in multiple scientific and industrial applications as varied as chemical sensing^1^, bioimaging and biosensing^2–4^, nanorulers^5^, advanced spectroscopy^6^ or surface-enhanced Raman spectroscopy^7^.

Plasmonic nanoparticles typically consist of nanometrical structures made of plasmonic materials such as noble metals (silver, gold, and platinum) or metamaterials that exhibit a negative real permittivity^8^. In this frame, conduction electrons performs a collective oscillation when they are excited by light at specific wavelengths, an effect known as localized surface plasmon resonance (LSPR). Plasmonic nanoparticles present better optical performance compared to standard fluorescence because they are optical emitters that exhibit very strong scattering while not being affected by quenching processes^9^.

The spectral fingerprint of plasmonic nanoparticles strongly depends on the shape, composition, dielectric environment, use of coatings and, above all, on their size^10,11^. This feature, makes them excellent sensors to detect small changes coming from the surrounding environment or inherent to the system itself, but in order to exploit it, it is necessary to have a very good control of the size and shape of the particles during the manufacturing process.

Most techniques used for the fabrication of gold nanoparticles (GNPs) are based on the reduction of gold chloride (HAuCl_4_) precursors applying various reducing agents, or on seeding growth methods. As seen in the literature, many different synthesis processes for GNPs have been developed^12–15^, yielding a large variety of nanoparticles with different sizes and shapes. However, a good control of the manufacturing process is still very challenging since there are some unsolved problems on how to achieve nanoparticles with a tight size distribution, and how to improve reproducibility between different nanoparticles lots^16–18^. Many GNPs are easily available on the market, but most of the time the size distribution can change significantly between different batches of the same vendor.

Given the difficulties found during the manufacturing processes, the need for accurate methods to measure their size is peremtory. Scanning Electron Microscopy (SEM) and Transmission Electron Microscopy (TEM) definitely represent the gold standard techniques to measure the size of nanoparticles^19–21^; the high spatial resolution achieved with these techniques (< 5 nm) allows visualizing any morphological nanoparticle features with excellent quality^22,23^.

Although SEM and TEM are widely used in many research laboratories and industries, there are some drawbacks when these experimental techniques are used: they need expensive instruments that work under vacuum conditions, require well-trained technicians and they are time-consuming, for both the sample preparation and measurement.

GNPs size can be also indirectly measured through optical methods; according to the work described by Haiss^24^, the optical absorbance of a colloidal solution measured with a standard spectrophotometer can be used to relate the optical absorbance peak with the nanoparticle diameter using Mie theory^25^. Although the method is fast and accurate, the main drawback is that the experimental data is inherently collected and averaged over a large distribution of particles. Optical absorbance spectra obtained from an ensemble of nanoparticles are always broadened and modified by their size distribution, their shape variability and by the agglomeration state of the colloidal solution.

In this paper, we introduce a new optical-based technique, named Dark-Field Single Particle Spectrophotometry (DF-SPS), that is able to measure the individual size of thousands of nanoparticles deposited onto a standard glass slide. Measurement of nanoparticle size is achieved by performing a spectral analysis of each individual nanoparticle standing on the surface. In analogy with a recent work^26^, spectral analysis of GNPs is performed by integrating the light scattered by the sample at each fixed wavelength and sweeping sequentially over the desired range of spectral components.

## Results and discussion

**Figure 1:**
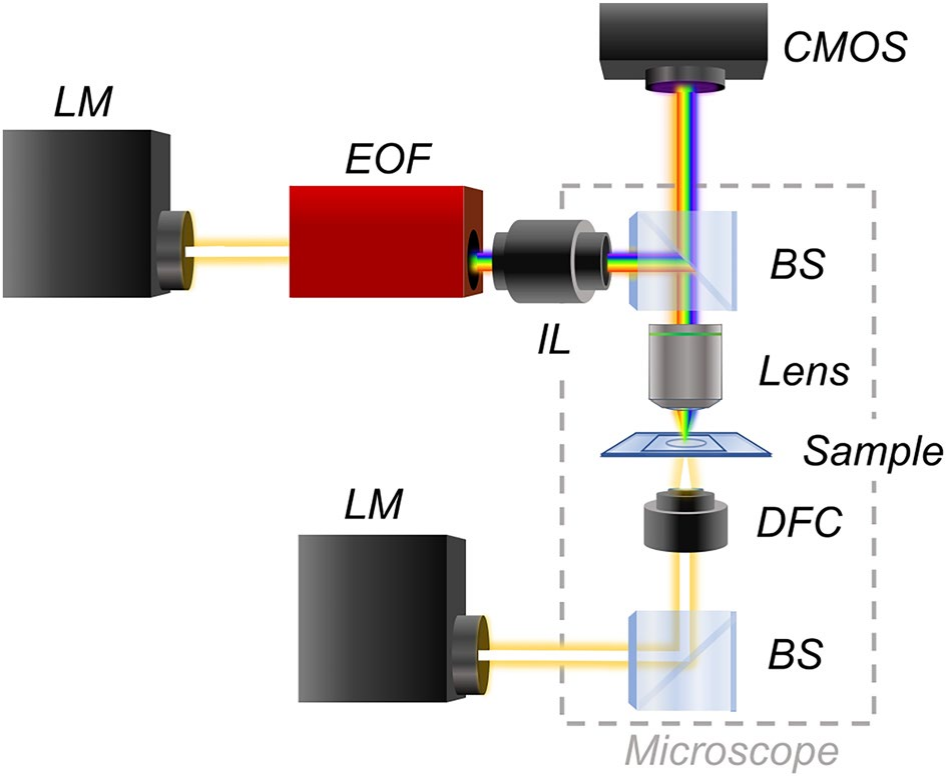
Schematic drawing of the customized optical instrument; the sample imaging is performed in transmission mode, while the sample spectrophotometry is done in reflection mode.

A schematic drawing of the experimental setup is shown in Fig. 1. The white light from a halogen lamp (LM) is directed to an electro-optical filter (EOF) that filters the light into its constituent wavelengths. The filtered spectral wavelengths coming from the tunable optical filter are then coupled to the epi-illuminator (IL) of the microscope and focused on the sample surface (SM) through a beam-splitter (BS) and a dark-field objective (OB). A CMOS camera placed at the image plane of the experimental setup collects the scattered light for each illumination wavelength. The optical microscope is equipped with a diascopic illuminator, thus in case of using transparent substrates such as in this work, it is able to perform imaging and micro spectrophotometry of the same sample region (more technical details are available in “Methods”). The DF-SPS technique is conceptually different compared to standard micro-spectrophotometry^27,28^. This is because spectral measurements on an extended sample area are performed in a parallel way without the needs of any scanning movements. The primary advantage of this technique is a much higher throughput that is at least one order of magnitude higher than state-of-the-art micro-spectrophotometry.

As a proof of concept, we assess the validity of the method by performing experiments on spherical gold nanoparticles (Nanopartz, Inc.) with seven different nominal sizes, dispersed at different concentration in milliQ® water. Before the experiments each nanoparticle lot was resuspended at a concentration of 200 µg/µl, wich is high enough for ensuring a good nanoparticle monodispersion but also suitable for achieving enough statistics with a single image capture (more informations are available in “Methods”).

**Figure 2:**
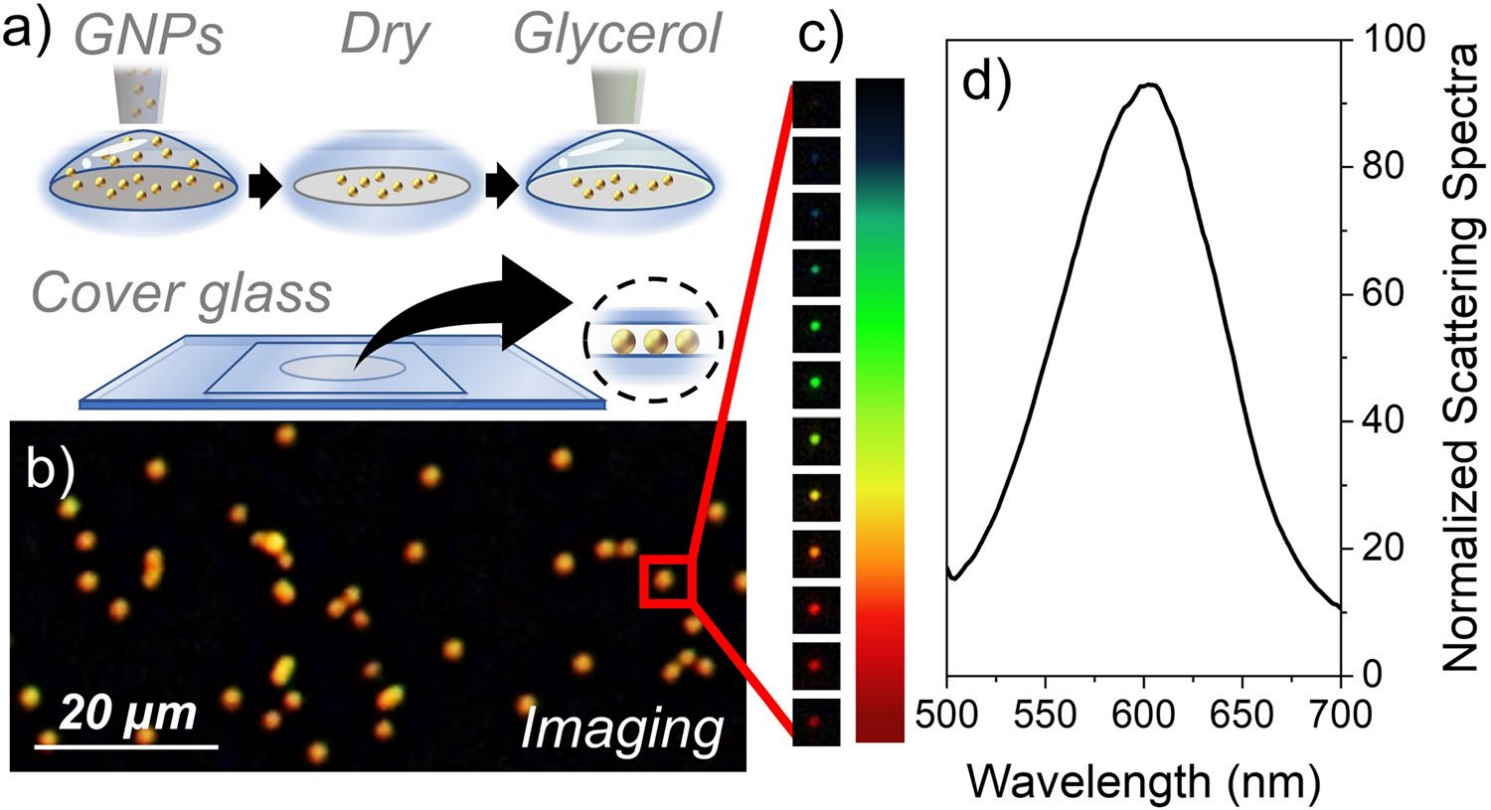
(**a**) Schematic drawing of the sample preparation. Nanoparticles are deposited onto a glass slide and then dried. Before adding a glass coverslip on top, a drop of glycerol is added to the dried nanoparticles. (**b**) Cropped dark-field image of nanoparticles deposited onto a transparent glass substrate; the image has been captured with the diascopic illuminator of the microscope in transmission mode. (**c**) Cropped dark-field image of a single nanoparticle taken at different wavelengths of the visible spectrum. (**d**) Scattering spectra of the nanoparticle identified by the red rectangular inset shown in (**b**); it has been normalized to the scattering signal coming from the substrate.

Nanoparticles were drop-casted on a glass slide, dried, and imbued in a drop of glycerol (Fig. 2a); the use of glycerol allows reducing the refractive index mismatch^29^ between the underneath glass substrate and the surrounding medium. In this way, nanoparticles are surrounded in a homogeneous medium, so splitting of substrate-mediated plasmonic modes is avoided^26,30^, and the optical response is well predicted by standard Mie theory^25,31^.

Before performing nanoparticle spectrophotometry, nanoparticles are imaged with the diascopic illuminator; a cropped dark-field image of 100 nm GNPs is shown in Fig. 2b; by using a proprietary algorithm based on the analysis of brightness and color of each individual optical spot present on the surface, it is possible to identify monomers from the rest of particles. One single image provides enough data to perform this analysis, allowing for fast measurement process (for more technical details, see the Supplementary Information 1).

Spectral measurements are then performed by measuring the scattering signal of the entire field of view at different spectral wavelengths. In a standard measurement, the complete 3D dataset is composed of 101 spectral images (from 450 to 650 nm with spectral steps every 2 nm) acquired in about 2 min. In order to eliminate any variations coming from pixel-to-pixel sensitivity of the detector and from distortions in the optical detection and illumination path, each dark-field image is flat-field corrected pixel-by-pixel according to the procedure described in the Supplementary Information.

**Figure 3:**
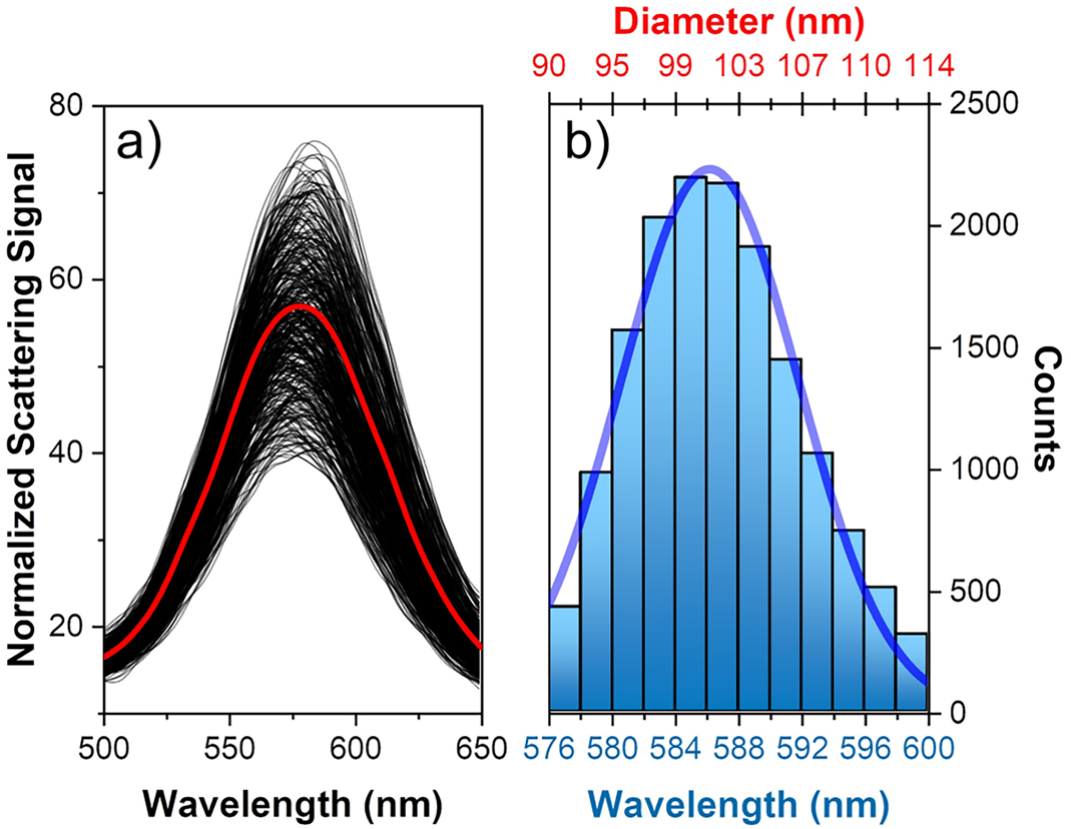
(**a**) Scattering spectra collected from more than 5000 monomers; the red curve represents the average of the scattering spectra of the entire monomer population. (**b**) Histogram of the plasmon resonance peak of 15,000 monomers shown in (**a**); the wavelength of the plasmon resonance peak has been obtained by performing a Lorentzian fitting of the scattering spectra shown in (**a**). The histogram’s top axis has been converted to nanoparticle diameter by making use of Eq. (1). The histogram data has been fitted to a Gaussian distribution, yielding an average size of 101.6 ± 2.8 nm and a CV around 6%.

A stack of dark-field images of a single monomer taken at different spectral wavelengths is shown in Fig. 2c. Scattering spectra of each monomer are calculated by integrating the wavelength-dependent scattering in a circular region around each monomer bound to the surface (Supplementary Information 2). The normalized scattering spectrum of a single 100 nm GNP shown in Fig. 2d results in good agreement with Mie scattering theory where a clear plasmon resonance peak around 575 nm is observed. For the sake of clarity, in this document normalized scattering spectra have been always obtained by dividing the scattering signal of each nanoparticle by the scattering signal coming from the substrate; this type of normalization allows obtaining a direct estimation of the signal to noise ratio for each measured nanoparticles. It is important to remark that all the detailed spectral features here illustrated for a single nanoparticle are also readily available for all other GNPs present within the field of view of the optical instrument. As in the current optical setup the entire field of view is about 0.55 mm^2^, resulting in an average nanoparticle density higher than 0.05 µm^2^, the DF-SPS is able to perform a spectral analysis of at least 5000 particles in a single shot. Remarkably, this number does not represent an upper limit for the DF-SPS; for example, by using an optical objective with a reduced optical magnification (for example, a 5× objective instead of the current 20×), this technique allows the measurements of up to 100,000 nanoparticles without any scanning movements. Scattering spectra collected from more than 15,000 monomers are shown in Fig. 3a; the red curve represents the average of the scattering spectra coming from the entire monomer population, showing an average resonance peak around 580 nm. The amplitude and the spectral variability observed in Fig. 3a are produced by the variability in size of the GNPs. It is remarkable that, although the GNPs present a very tight size distribution (CV < 5%), the amplitude variability is significantly higher (CV around 30%), in good agreement with Mie scattering theory.

The spectrum of each single monomer has been fitted with a Lorentzian peak, allowing to determine the wavelength of the plasmon resonance peak with an uncertainty below 1 nm (more details about data analysis can be found in the Supplementary Information 3). The histogram of the plasmon resonance peaks of 15,000 monomers is shown in Fig. 3b. For GNPs, the relationship between the nanoparticle size and the wavelength of the plasmon resonance peak can be well approximated with a simple logarithmic function:

1 $${\text{d}} \cong \frac{{\text{1}}}{{{\text{C}}_{{\text{2}}} }}{\text{ln}}\left( {\frac{{{{\lambda }} - {{\lambda }}_{{\text{0}}} }}{{{\text{C}}_{{\text{1}}} }}} \right)$$

where ${\lambda}_{0}$, ${\text{C}}_{1}$ and ${\text{C}}_{2}$ are constants whose values depends on the optical properties of the nanoparticle material; in case of gold ${\lambda}_{0}= \text{530 nm}$, ${\text{C}}_{1}\text{=} \, \text{6.53 nm}$ and ${\text{C}}_{2}\text{=} \, \text{0.0216 }{\text{nm}}^{-{1}}$.

By making use of Eq. (1), the top axis of Fig. 3b has been converted to nanoparticle diameter; this conversion is extremely useful because it provides a quick and direct estimation of the nanoparticle size based on its plasmon resonance peak. The histogram data of Fig. 3b has been fitted with a Gaussian function^32^, yielding an average size of 101.6 nm ± 2.8 nm and a CV around 6%; the values found here are in very good agreement with the technical specifications given by the manufacturer (nominal size and CV of 100 nm and 4%, respectively).

**Figure 4:**
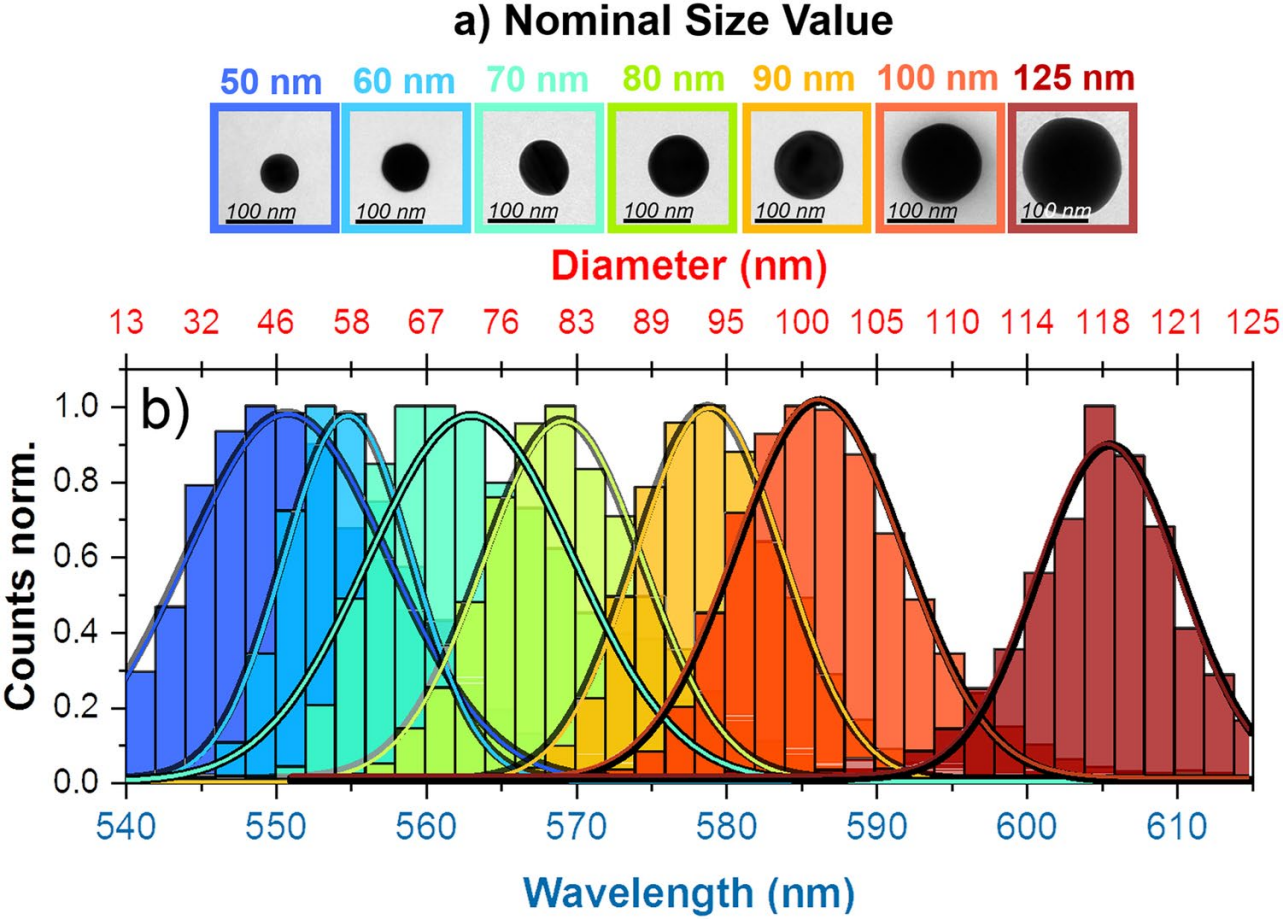
(**a**) TEM images of monomers with different nominal size. (**b**) Histogram distributions of the plasmon resonance peak of all seven nanoparticle lots characterized; the histogram’s top axis has been converted to nanoparticle diameter by making use of Eq. (1). Each histogram has been fitted with a Gaussian distribution (solid line).

Following the same procedure described previously for 100 nm nanoparticles, other nanoparticle lots with different nominal size have been measured; in total, seven different lots with diameters ranging from 50 to 125 nm have been studied (representative scattering spectra with Lorentzian fits of all the nanoparticle batches measured are reported in the Supplementry Information 4); for this study, we have tested all the nanoparticles sizes that available from the GNPs provider. TEM images shown in Fig. 4a confirm that, except for the 70 nm GNP lot that has a more pronounced elliptical shape, all others characterized lots present a high spherical shape. (more technical details about TEM measurmeents and characterization can be found in the Supplementary Information 7). All histogram distributions of the plasmon resonance peak for each nanoparticle lot are summarized in Fig. 4b; the histogram’s top axis of Fig. 4b has been converted to nanoparticle diameter by using Eq. (1). For each lot, at least 5000 GNPs monomers have been characterized. The histogram corresponding to each lot has been fitted with a Gaussian distribution. Although the estimated diameters and their CV tightly match the nominal values given by the manufacturer, a significant deviation from a Gaussian distribution is observed in the case of 70 nm; while the rest of the lots have an R-squared value of ~ 0.99, for 70 nm this parameter is ~ 0.95 (the closer the fit is to the data points, the closer R-squared will be to 1). As mentioned above, the lower roundness of this nanoparticle lot could explain the increased deviation.

**Figure 5:**
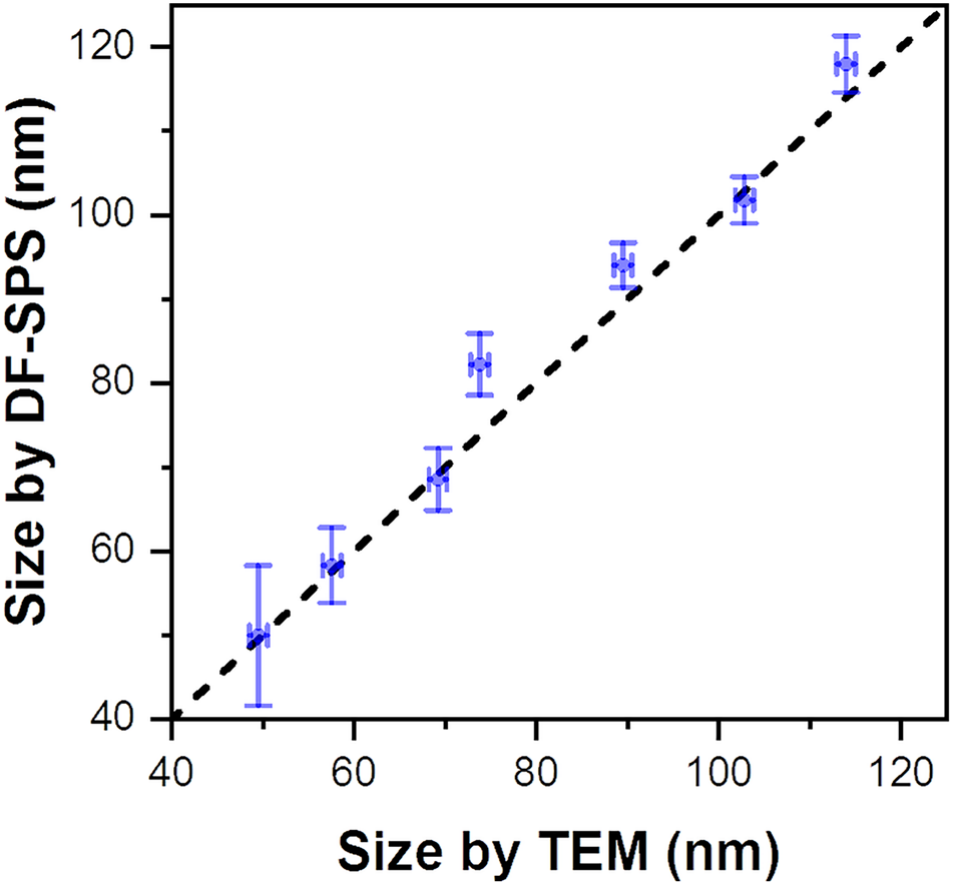
Correlation graph between GNP sizes measured with TEM and estimated with DF-SPS. The dashed red line in the graph represents a perfect linear relationship.

In order to check the accuracy of the DF-SPS technique in the diameter estimation, a direct comparison with TEM is needed. The graph in Fig. 5 shows the correlation between the real diameter measured with TEM and the diameter estimated with DF-SPS. The error bars in X and Y are the standard deviations coming from TEM and DF-SPS, respectively; for TEM and DF-SPS at least 500 and 5000 per batch have been characterized. The correlation graph presents excellent agreement between TEM and DF-SPS, with a mean discrepancy around 3% (additional compared data between TEM and DF-SPS can be found in the Supplementaty Information 8). Taking into account all seven characterized lots, the resulting Pearson’s correlation coefficient between the sizes estimated with both techniques is 0.9904, indicating a dependence very close to a perfect linear relationship. Although the feasibility of the method has been here proved by using spherical nanoparticles, the true strengthness of the method is that it can be easily generalized also to nanoparticles with more complex geometries such as nanorods, nanocubes, nanoplates or core–shell geometries^33^.

**Figure 6:**
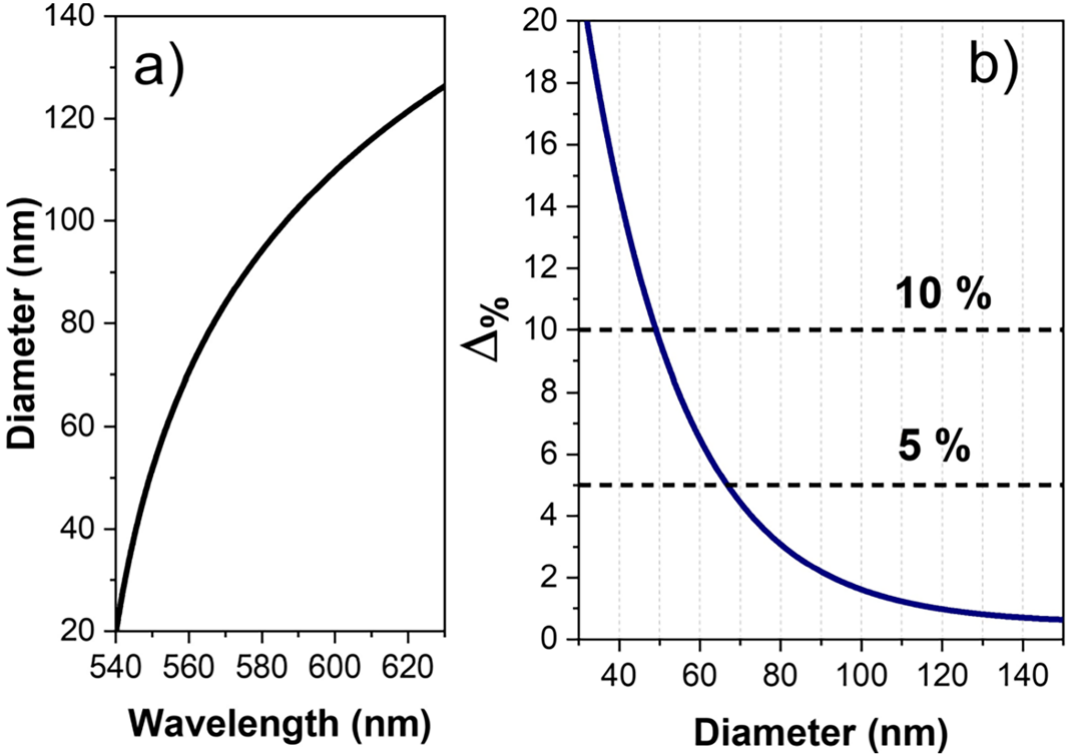
(**a**) Theoretical dependence of Eq. (1) that establishes the dependence between the nanoparticle diameter and the wavelength of the plasmon resonance peak. (**b**) Theoretical percentage uncertainty in the estimation of the nanoparticle diameter with DF-SPS.

Remaining now to the specific case of spherical nanoparticles, we will analyze in the following how the size accuracy tends to be lower for particles smaller than 40–50 nm. This aspect becomes clear once Eq. (1) is analyzed and plotted (see Fig. 6a). For nanoparticles smaller than 40 nm, the wavelength of the plasmon resonance peak remains almost constant, thus producing a significant uncertainty in the diameter estimation.

The percentage uncertainty of the DF-SPS can be obtained using the following equation:

2 $$\Delta _{{{\% }}} \;{{ = }}\;{{100~}}\frac{{\partial {\text{d}}}}{{\partial {{\lambda }}}}\frac{{\Delta {{\lambda }}}}{{\text{d}}}$$

where $${\raise0.7ex\hbox{${\partial {\text{d}}}$} \!\mathord{\left/ {\vphantom {{\partial {\text{d}}} {\partial {{\lambda }}}}}\right.\kern-\nulldelimiterspace} \!\lower0.7ex\hbox{${\partial {{\lambda }}}$}}$$ is the first derivative of Eq. (1), d is the nanoparticle diameter and $$\Delta {{\lambda }}$$ is the wavelength uncertainty that in the current experimental setup is around 1 nm (more technical details about the derivation of Eqs. (1) and (2) can be found in the Supplementary Informations 5 and 6).

The percentage uncertainty curve (Fig. 6b) presents a significant diameter-dependence; for instance, nanoparticles of 100 nm feature an uncertainty below 2%. As the particle size decreases, the uncertainty progressively increases: an uncertainty below 5% can be reached for particles of 70 nm, whereas nanoparticles of 50 nm present a percentage uncertainty of around 10%. It is important to remark, that the limitation here observed for smaller nanoparticles does not arise from experimental error but uniquely from a limitation imposed by theory; although, scattering signal is significantly smaller by reducing the nanoparticle size, the measured signal to noise ratio even for the smallest nanoparticle size is still very high (for more information, see more details in Supplementary Information 4).

Although in the present proof-of-concept experiment high quality spherical nanoparticles have been used, in many real situations synthesized nanoparticles differ from ideal spherical shape. Non-spherical nanoparticles could slightly deviate from Eq. (1) and a deeper spectral analysis is required; for instance, there are many spectral fingerprints that could be measured and analyzed with DF-SPS such as the broadening measurement of the plasmon resonance linewidth^34^, the fitting of the spectral response with multiple Lorentzian fittings^35^, or the measurement of the correlation between the scattering amplitude and the wavelength of the plasmon resonance peak^36^.

As DF-SPS is perfectly suitable also for the spectral characterization of nanoparticles with more complex geometries, we are currently working on the characterization of gold nanorods with variable aspect ratio (no data shown). As it is well known in literature^37,38^, nanorods present two narrow plasmon resonances that are associated to the collective electron excitations along the short and long axis of the particle; by measuring the wavelength of the two plasmon resonance peaks, both short axis, long axis and their aspect ratio of individual nanorods could be promptly quantified with the DF-SPS.

## Conclusions

In this work we present a new optical method, named Dark-Field Single Particle Spectrophotometry, able to measure the individual size of thousands of particles with nanometrical accuracy and in only a few minutes. Sample preparation is simple and based on drop-casting on top of a glass slide, while the experimental setup is based on a customization of a standard optical micro-spectrophotometer. As a proof of concept, spherical nanoparticles with different sizes have been measured and after a direct comparison with Transmission Electron Microscopy (TEM), a mean discrepancy around 3% has been demonstrated. Although the feasibility of the method has been here proved with spherical nanoparticles, the true strengthness of the method is that it can be also generalized to nanoparticles with arbitrary shapes or even to core–shell geometries^33^.

Thanks to the simplicity, and the high performance in terms of size accuracy and throughput, this novel method opens new routes in metrological characterization of plasmonic particles because it can be easily implemented in any research or synthesis laboratory, thus representing an advantageous alternative to the gold-standard electron microscopy.

## Methods

### Experimental set-up

The practical realization of the microspectrophotometer is achieved by using a 50 W halogen lamp and a VI-IR electro-optical filter (*Thorlabs Kurios WB1*, *Thorlabs*, working spectral range from 400 to 700 nm) coupled through a mechanical adapter to the epi-illuminator arm of a commercial optical microscope (*Nikon Eclipse Ni-U*) and a dark-field optical objective (20×, NA 0.4, MUE 61200 from Nikon). Measurements were done with a scientific-grade CMOS monochromatic camera (*Ximea MC050MG-SY*). As conventional dark-field objectives are usually designed only for dry conditions, once that they are used with samples covered with a coverslip, chromatic and spherical aberrations could be introduced. However this effect can be considered negligible for dry objectives with low numerical aperture^39^ such as in the current case (NA = 0.4).

The instrument is also able to perform microscopy measurements by using a diascopic illuminator composed of a 100 W halogen lamp coupled to a dark-field condenser; microscopy measurements are acquired with an RGB CMOS camera (*Nikon DS-Fi 2*). Both monochromatic and color cameras are simultaneously mounted along the detection path of the instrument by using a dual port (*Y-IDP dual port from Nikon*). Most of the hardware components of the instruments (RGB and monochromatic cameras, tunable optical filter, halogen lamps) are controlled by software developed in-house.

### Sample preparation

The experiments were performed with spherical gold nanoparticles from *Nanopartz* with seven different nominal sizes: 50, 60, 70, 80, 90, 100 and 125 nm. As each nanoparticle lot is initially dispersed in milliQ® water at a concentration in the mg/mL range, nanoparticles are resuspended in milliQ® water at a concentration of 200 µg/µL; this range of concentration ensures both good nanoparticle monodispersion and sufficiently good statistics with a single image capture. In order to improve the nanoparticle monodispersity, the colloidal solution is sonicated for 5 min and vortexed for 5 min prior to sample preparation. A drop of 50 µL was deposited onto a transparent glass slide (*Thermo Fisher, Microscope slides, Menzel Gläser*) and dried under ambient temperature conditions. Before the addition of the glass coverslip (*Menzel Deckgläser 20* × *20 mm, 170 µm thick*) on top of the sample substrate, a droplet of 2 µL of glycerol (*Sigma Aldrich*) is added to the dried GNPs. The addition of glycerol reduces the optical mismatch between the refractive index of the glass substrate and that of the surrounding environment.

In this way, nanoparticles are surrounded in a homogeneous medium, so no splitting of substrate-mediated plasmonic modes is observed and the optical response is well predictable by standard Mie theory.

## Supplementary Information

Supplementary Information.

## Data Availability

All details about monomers detection from images, data processing and analysis and experimental TEM and DF-SPS characterization procedures, methods and data are provided in the text and Supplementary Information. Any clarifications will be avaible by contacting the corresponding author.
