# Supplementary material for: Size characterization of plasmonic nanoparticles with dark-field single particle spectrophotometry
Source: Sci Rep. 2022 Oct 24;12:17231. doi: 10.1038/s41598-022-21649-8 (PMC9592611; doi:10.1038/s41598-022-21649-8)
Supplement: Supplementary file 1 — Supplementary Information. [file 41598_2022_21649_MOESM1_ESM.docx]

**Supplementary information**

**Size characterization of Plasmonic Nanoparticles with Dark-Field Single Particle Spectrophotometry**

**Rodrigo Calvo^1,2^, Andreas Thon^1^, Asis Saad^1^, Antonio Salvador-Matar^1^, Miguel Manso Silván^2^, Óscar Ahumada^1^, Valerio Pini^1,*^**

1Mecwins, Roda de Poniente 15, Tres Cantos, 28760, Madrid, Spain

2Departamento de Física Aplicada, Universidad Autónoma de Madrid, Campus de Cantoblanco, 28049, Madrid, Spain

*[vpini@mecwins.com](mailto:vpini@mecwins.com)

1. **Monomers Detection from image analysis**

In each image taken with the DF-SPS there are multiple nanoparticles, and in many cases the GNPs form agglomerations, such as dimers, trimers, or larger clusters. As nanoparticle agglomerations present very complex spectra which are difficult to model theoretically, they have been discarded in the following analysis. Before performing the spectral analysis of the sample, a color image of the sample is taken; the captured color image allows a rapid identification and classification of the monomers and their positions within the sample according to the procedure described below.

The particle recognition software first identifies potential particles based on their shape and size. A gray-scale test pattern is generated which represents the typical shape of a nanoparticle in an image from the camera, considering the actual image acquisition parameters used. Here, the shape of a nanoparticle is represented by a radially symmetric two-dimensional Gaussian function. The normalized two-dimensional cross-correlation between the grayscale image and the test pattern is calculated. This image has values close to "1" for regions that are similar in shape to the test pattern. Local maxima, *i.e.*, pixels with values higher than those of all their direct neighbors, are located and the positions are considered as potential positions of the particles. Once detected, the position and average brightness of each particle is stored, as well as the values of the three RGB channels.

The discrimination of particles is based on the amount of scattering and on the normalized values of one color component; in the example, the red component is used. Both values form the axes of a 2-dimensional histogram in which the particle types are identified: noise, dust, monomers (individual GNPs), dimers, trimers, clusters etc. (Figure S1).


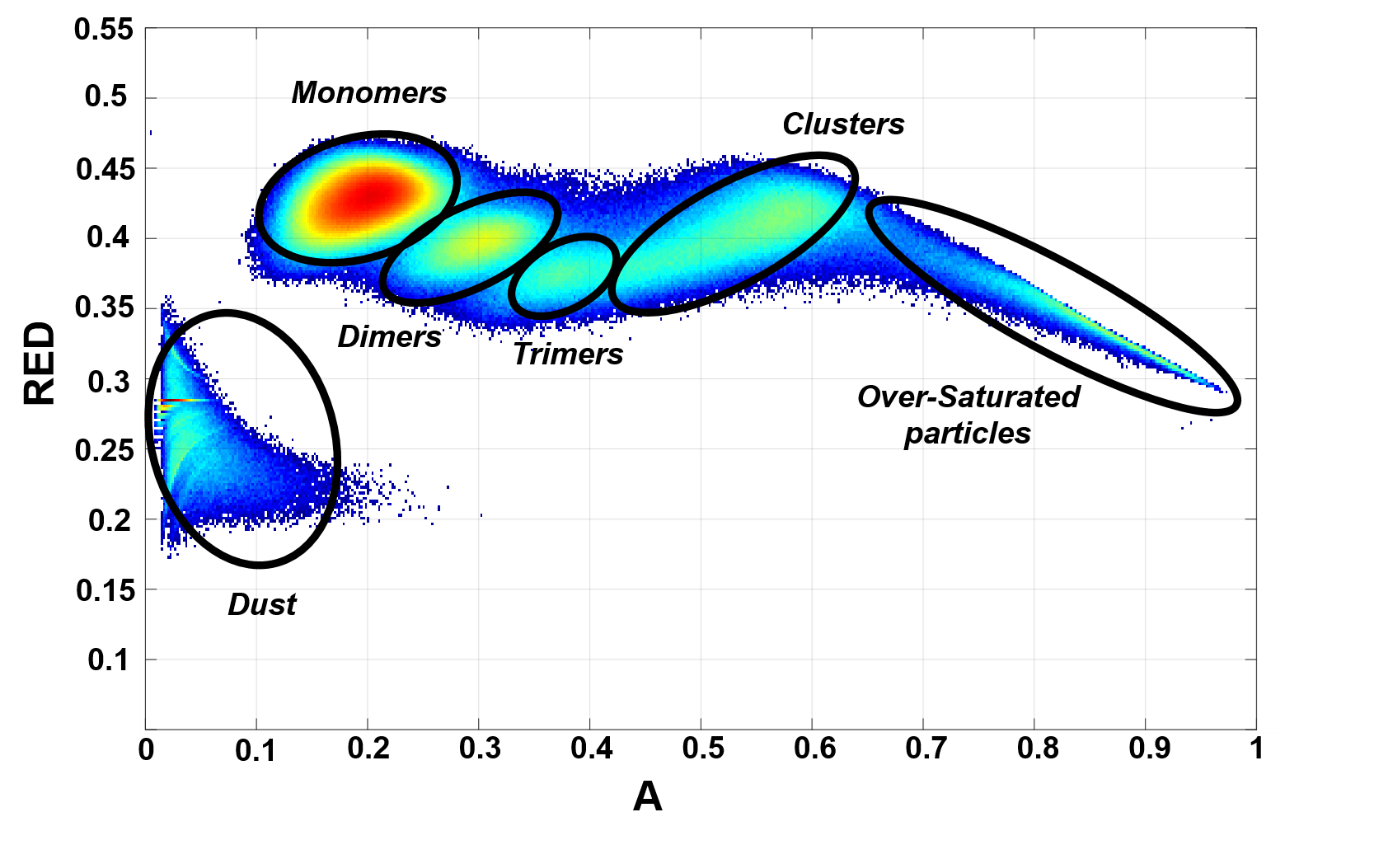


**Figure S1**: Color-coded 2D histogram of the brightness and the red channel of 100 nm detected particles (reddish colors indicate higher counts, bluish colors lower counts); both brightness and red channels have been normalized to the maximum allowable value. Each type of particles can be easily identified and distinguished in 2D histogram.

Once the particles are classified, the number of particles in each class is counted, and the location of each particle in the image is stored. After this identification and classification procedure, each monomer present on the surface can be identified in the image with a pair a XY coordinates.

1. **Data processing**

During the spectral measurement of the sample surface, a tridimensional dataset commonly known as spectral cube is generated. The spectral cube is obtained by stacking the scattering signal of the entire field of view at different spectral wavelengths; so, in this way, the first two dimensions of the spectral cube represent the sample surface, while the third one corresponds to the wavelength. In a standard spectral measurement, the complete 3D dataset is composed of 101 spectral images (from 450 nm to 650 nm with steps of 2 nm). Each spectral image is a monochromatic picture of 5 MP with a bit depth of 88, i.e., the brightness scale per pixel then runs from 0 to 255. As the monochromatic images are saved during the measurement in TIFF format, before the creation of the spectral cube the images are converted to a linear brightness representation, by eliminating the non-linear compression coming from the gamma encoding algorithm.^1^

In order to eliminate any variations coming from pixel-to-pixel sensitivity of the detector and by distortions in the optical detection and illumination path, each monochromatic image is also flat-field corrected pixel-by-pixel using the following equation:

| $\text{I}_{\text{C}}\text{=m }\frac{\text{I}_{\text{R}}\text{-DF}}{\text{I}_{\text{F}}\text{-DF}}$ | (1) |
| --- | --- |

where $\text{I}_{\text{C}}$ is the corrected image, $\text{I}_{\text{R}}$ is the raw image, $\text{DF}$ is the dark frame, and $\text{I}_{\text{F}}$ is the flat frame, while m is a constant defined as the averaged value of the difference image $\text{I}_{\text{F}}-DF$. For the flat frame, a diffuse reflectance standard has been used (*SRS-99-010 from Labsphere*).

1. **Data analysis**

Once the 3D dataset has been linearized and flat-field corrected, scattering spectra of each individual monomers are calculated. According to the previous classification described in section 1, all the XY coordinates of each individual monomer are well known, and the scattering size of each monomer is defined by the optical resolution of the objective and the spatial resolution of the detector used (in the current setup each particle is defined by a circular area of 25 pixels).

Scattering spectra of each monomer are calculated by integrating the wavelength-dependent scattering in a circular region around each XY monomer’s coordinate. To each of these spectra, a Lorentzian fitting is adjusted:

| $\text{S}\left( \text{λ} \right)\text{ =}\frac{\text{2 A}}{\text{π}}\frac{\text{w}}{\text{4}{\text{(λ-}\text{λ}_{\text{SPS}}\text{)}}^{\text{2}}\text{+}w^{2}}$ | (2) |
| --- | --- |

where S(λ) is the wavelength-dependent scattering signal of the particle, $\text{A}$ the curve amplitude, w is the peak’s width, and $\text{λ}_{\text{SPS}}$ the wavelength of the plasmon resonance peak.

After the fitting procedure, a filtering process based on the coefficient R-squared (R^2^ > 0.9) is performed; this filtering process allows discarding the few unwanted particles that may have been erroneously identified and classified as monomers.

1. **Representative scattering spectra and fittings for different GNPs sizes**

The representative spectra and the Lorentzian fits for all the nanoparticles batches measured in this work are shown in Figure S2; all the scattering spectra have ben normalized by the scattering signal of the substrate, giving a direct estimation of the signal to noise ratio that we have for each type of nanoparticle. Although scattering signal is significantly smaller by reducing the nanoparticle size, also in case of the smallest 50 nm nanoparticles, the signal to noise ratio is around 10, a value that allows performing a good fitting without losing accuracy.


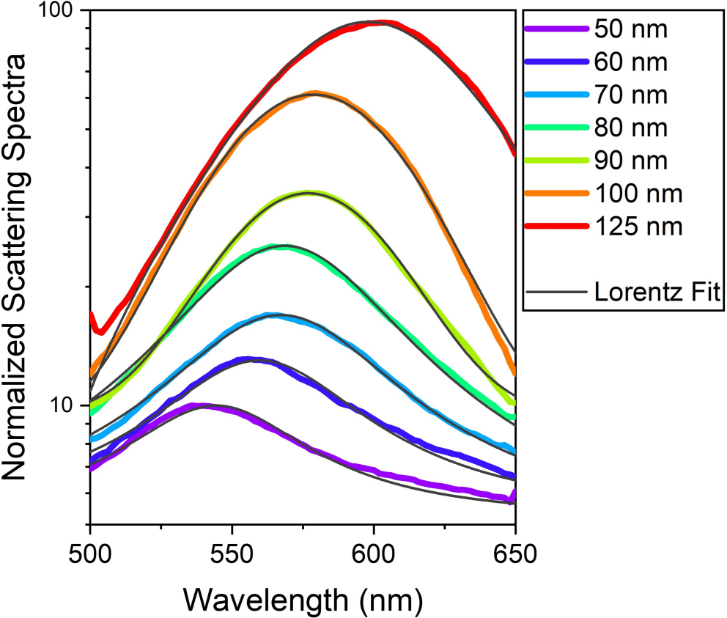


**Figure S2**: Representative spectra and their relative Lorentzian fits (solid light gray) for all the nanoparticles batches measured in this work; all the scattering spectra have ben normalized by the scattering signal of the substrate, giving a direct estimation of the signal to noise ratio that we have for each type of nanoparticle. As the light scattering drastically change with the nanoparticle size, the normalized scattering signals are plot in logarithmic scale.

1. **Mie theory**

Mie theory describes the scattering cross section^2,3^ of a spherical particle of radius *r* inside a medium with a dielectric function $\epsilon_{m}$ at a given wavelength:

| $\text{σ}_{\text{sca}}\text{=}\frac{\text{λ}^{\text{2}}}{\text{2π}}\sum_{\text{l=1}}^{\text{∞}} \text{(2n+1)(}\left\vert\text{a}_{\text{l}} \right\vert^{\text{2}}\text{+}\left\vert\text{b}_{\text{l}} \right\vert^{\text{2}}\text{)}$ | (3) |
| --- | --- |

$a_{l}$ and $b_{l}$ are the scattering coefficients in terms of the Ricatti-Bessel spherical functions $\text{η}_{\text{l}}\left( \text{x} \right)$

and $\text{ψ}_{\text{l}}\left( \text{x} \right)$

| $\text{a}_{\text{l}}\text{ = }\frac{\text{m}\text{ψ}_{\text{l}}\left( \text{mx} \right)\text{ψ}_{\text{l}}^{\text{'}}\left( \text{mx} \right)\text{-}\text{ψ}_{\text{l}}^{\text{'}}\left( \text{mx} \right)\text{ ψ}_{\text{l}}\left( \text{mx} \right)}{\text{m}\text{ ψ}_{\text{l}}\left( \text{mx} \right)\text{η}_{\text{l}}^{\text{'}}\left( \text{x} \right)\text{-}\text{ψ}_{\text{l}}^{\text{'}}\left( \text{mx} \right)\text{η}_{\text{l}}\left( \text{x} \right)}$ | (4) |
| --- | --- |
| $\text{b}_{\text{l}}\text{ = }\frac{\text{ ψ}_{\text{l}}\left( \text{mx} \right)\text{ψ}_{\text{l}}^{\text{'}}\left( \text{x} \right)\text{-m}\text{ψ}_{\text{l}}^{\text{'}}\left( \text{mx} \right)\text{ ψ}_{\text{l}}\left( \text{x} \right)}{\text{ ψ}_{\text{l}}\left( \text{mx} \right)\text{η}_{\text{l}}^{\text{'}}\left( \text{x} \right)\text{-}\text{mψ}_{\text{l}}^{\text{'}}\left( \text{mx} \right)\text{η}_{\text{l}}\left( \text{x} \right)}$ | (5) |

Here, $\text{x=}\frac{\text{2πr}}{\text{λ}}$ is the size parameter defined as the ratio between the nanoparticle’s characteristic dimension $r$ and the light wavelength $\text{λ}$ and $\text{m=n(r)/}\text{n}_{\text{m}}$ is the ratio between the refractive index of the particle $\text{n}$ and the surrounding medium $\text{n}_{\text{m}}$. In this experiment, the surrounding medium is glycerol so $\text{n}_{\text{m}}\text{=1.47}$. For the numerical calculation of the scattering signal, Mie functions based on Mätzler’s (2022) code^4^ have been used.

Optical properties of particles with a size comparable to the mean free path of conduction electrons ($\text{l}_{\text{∞}}^{\text{gold}}\text{\textasciitilde40nm}$) present significant variations compared to the optical properties of bulk materials. According to the extended Drude model,^5^ the collision of free electrons with the surface become even more important for smaller nanoparticle sizes; due to this, also the collision frequency results size-dependent:

| $\text{ω}_{\text{0}}\left( \text{r} \right)\text{ =}\text{ ω}_{\text{0}}\text{+}\text{υ}_{\text{F}}\text{/r}$ | (6) |
| --- | --- |

where $\text{ω}_{\text{0}}$ is the collision frequency for bulk material and $\text{υ}_{\text{F}}$ is the Fermi velocity; for gold $\text{ω}_{\text{0}}\text{=3.4·}\text{10}^{\text{13}}\text{ }\text{s}^{\text{-1}}$ and $\text{υ}_{\text{F}}\text{=1.4·}\text{10}^{\text{6}}\text{ m/s}$.

The dielectric constant in metals can now be written in this way:

| $\text{ε}\left( \text{λ,r} \right)\text{=}\text{(A}_{\text{1}}\left( \text{r,λ} \right)\text{+}\text{B}_{\text{1}}\text{(λ))+}\text{i}\text{(A}_{\text{2}}\left( \text{r,λ} \right)\text{+}\text{B}_{\text{2}}\text{(λ))}$ | (7) |
| --- | --- |

where $\text{A}_{\text{1}}\left( \text{r,λ} \right)$ and $\text{A}_{\text{2}}\left( \text{r,λ} \right)$ consist of an additive contribution from free electrons

| $\text{A}_{\text{1}}\left( \text{r,λ} \right)\text{ = 1-}\frac{\text{ω}_{\text{P}}^{\text{2}}}{\left( \text{λ/c} \right)^{\text{2}}\text{+}\text{ω}_{\text{0}}^{\text{2}}\text{(r)}}$ | (8) |
| --- | --- |
| $\text{A}_{\text{2}}\left( \text{r,λ} \right)\text{ = }\frac{\text{ω}_{\text{P}}^{\text{2}}\text{ω}_{\text{0}}\left( \text{r} \right)}{{\frac{\text{λ}}{\text{c}}\text{[}\left( \text{λ/c} \right)}^{\text{2}}\text{+}\text{ω}_{\text{0}}^{\text{2}}\text{(r)]}}$ | (9) |

While $\text{B}_{\text{1}}\text{(λ)}$ and $\text{B}_{\text{2}}\text{(λ)}$ are two terms related to the bound electrons that can be calculated by using the optical properties of bulk material; for the calculation of the spectral response of gold nanoparticles, optical properties from Johnson and Christy database have been used^6^_._ $\text{ω}_{\text{p}}\text{=2.369·}\text{10}^{\text{15}}\text{ }\text{s}^{\text{-1}}$ is the plasma frequency for gold.

The refractive index $\text{n}\left( \text{r,λ} \right)$ and the extinction coefficient $\text{k}\left( \text{r,λ} \right)$ of the GNPs can now be calculated by using the following equation:

| $\text{n}\left( \text{r,λ} \right)\text{;k}\left( \text{r,λ} \right)\text{ =}\left[ \text{±}\frac{\text{A}_{\text{1}}\left( \text{r,λ} \right)\text{+}\text{B}_{\text{1}}\text{(λ)}}{\text{2}}\text{+}\left( \left( \frac{\text{A}_{\text{1}}\left( \text{r,λ} \right)\text{+}\text{B}_{\text{1}}\text{(λ)}}{\text{2}} \right)^{\text{2}}\text{+}\left( \frac{\text{A}_{\text{2}}\left( \text{r,λ} \right)\text{+}\text{B}_{\text{2}}\text{(λ)}}{\text{2}} \right)^{\text{2}} \right)^{\text{1/2}} \right]^{\text{1/2}}$ | (10) |
| --- | --- |

By making use of equation (3), a relationship between the wavelength of the plasmonic peak and the nanoparticle size can be derived. This relationship can be well approximated with a simple exponential function:

| $\text{d}\text{ }\text{≅}\text{ }\frac{\text{1}}{\text{C}_{\text{2}}}\text{ln}\left( \frac{\text{λ-}\text{λ}_{\text{0}}}{\text{C}_{\text{1}}} \right)$ | (11) |
| --- | --- |

where d is the nanoparticle diameter while $\text{λ}_{\text{0}}$, $\text{C}_{\text{1}}$ and $\text{C}_{\text{2}}$ are fitted parameters whose value depends on the optical properties of the nanoparticle material: in case of gold, $\text{λ}_{\text{0}}\text{=530 nm}$, $\text{C}_{\text{1}}\text{=6.53 nm}$ and $\text{C}_{\text{2}}\text{=0.0216 }\text{nm}^{\text{-1}}$.

1. **Error and uncertainty propagation in size calculations**

With $\text{λ}_{\text{0}}$, $\text{C}_{\text{1}}$ and $\text{C}_{\text{2}}$ being constants, this implies that an uncertainty in the determination of the peak wavelength, will cause an uncertainty in the diameter of the particles derived with equation (11). In a linear approximation, and here for the case of a single independent variable, x, the propagation from an uncertainty $\sigma_{x}$ to an uncertainty $\sigma_{y}$ via function $y=f(x)$ is given by:^7^

| $\sigma_{y}\text{=}\sqrt{\left( \frac{\partial f}{\partial x} \right)^{2}\cdot\sigma_{x}^{2}}=\left\vert\frac{\partial f}{\partial x} \right\vert\cdot\sigma_{x}$ | (12) |
| --- | --- |

The linear approximation in valid if the function $f(x)$ in a range defined by the uncertainty $\sigma_{x}$ can be well approximated with a linear function. Applied to the function $d(\lambda)$ used here, we obtain the partial derivative

| $\frac{\partial d}{\partial\lambda}=\frac{\partial}{\partial\lambda}\left( \frac{1}{C_{2}}\ln\left( \frac{\lambda-\lambda_{0}}{C_{1}} \right) \right)=\frac{1}{C_{2}}\cdot\frac{1}{\frac{\lambda-\lambda_{0}}{C_{1}}}\cdot\frac{1}{C_{1}}=\frac{1}{C_{2}\left( \lambda-\lambda_{0} \right)}$ | (13) |
| --- | --- |

The uncertainty in the derived diameter $d(\lambda)$ is thus:

| $\sigma_{d}\text{=}\left\vert\frac{\partial d}{\partial\lambda} \right\vert\cdot\sigma_{\lambda}=\left\vert\frac{1}{C_{2}\left( \lambda-\lambda_{0} \right)} \right\vert\cdot\sigma_{\lambda}=\frac{\sigma_{\lambda}}{C_{2}\left\vert\lambda-\lambda_{0} \right\vert}$ | (14) |
| --- | --- |

Expressed as a relative uncertainty, in percent, the uncertainty is:

| $\Delta_{\%}=100\%\cdot\frac{\sigma_{d}}{d}=\mathbf{100}\boldsymbol{\%}\boldsymbol{\cdot}\left\vert\frac{\boldsymbol{\partial}\mathbf{d}}{\boldsymbol{\partial\lambda}} \right\vert\boldsymbol{\cdot}\frac{\boldsymbol{\sigma}_{\boldsymbol{\lambda}}}{\mathbf{d}}$ | (15) |
| --- | --- |
| $=100\%\cdot\frac{\sigma_{\lambda}}{C_{2}\left\vert\lambda-\lambda_{0} \right\vert}\cdot\frac{1}{d}=100\%\cdot\frac{\sigma_{\lambda}}{\left\vert\lambda-\lambda_{0} \right\vert\ln\left( \frac{\lambda-\lambda_{0}}{C_{1}} \right)}$ | (16) |

the equation (15) is the one used as equation (2) in the manuscript.

As commented before, for this calculation it has been assumed that the function $f(x)$ can be well approximated with a linear function in a range defined by the uncertainty $\sigma_{x}$. Here, $\sigma_{x}$ is the uncertainty of the determination of the peak wavelength, which has been assumed to be $\sigma_{\lambda}=1 nm$ because of the experimental setup. To make sure that the linear approximation in the error propagation is valid, the exact values of $d(\lambda\pm\sigma_{\lambda})$ have been compared with the values obtained using a linear approximation, $d(\lambda)\pm\cdot\sigma_{\lambda}$. For $\sigma_{\lambda}=1 nm$, the error caused by the linear approximation is at least a factor of 20 smaller than the relative uncertainty of the derived diameter $d(\lambda)$, which justifies the use of the linear approximation in the error propagation.

1. **TEM measurements and characterization**

The inspection of plasmonic nanoparticles with TEM allows determining the real size and shape of the GNPs and comparing it with the nominal value specified by the manufacturer.

Nanoparticles have been characterized by using a high-voltage transmission electron microscope (*JEOL JEM1010*). The electron beam voltage has been set to 100kV for all the measurements, and in order to ensure images with high resolution, the magnification has been set to 300kX. For each nanoparticle lot, at least 500 individual nanoparticles have been characterized, ensuring a good statistical distribution of nanoparticle sizes and shapes.

In order to measure the nanoparticle diameter, a custom software has been developed that consists in detecting spherical objects with an algorithm based on the circular Hough Transform (CHT)^8^. This method allows evaluating the nanoparticle diameter with an uncertainty lower than 1%.

The histogram distributions of the diameters of each nanoparticle lot are shown in Figure S3. As summarized in Table S1, TEM analysis confirms that the real size of each GNPs lot is in very good agreement with the nominal value specified by the manufacturer, the mean discrepancy between the real size and the nominal one being below 3.5 %.

Hough transform can also detect the degree of ellipticity of each nanoparticle, allowing us to calculate their aspect ratio according to the following equation:

| $\rho\text{ = }\frac{{major}_{ax}}{{minor}_{ax}}$ | (17) |
| --- | --- |

where ${major}_{ax}$ and ${minor}_{ax}$ are the major and the minor axes, respectively. As it is summarized in Table S2, the measured NPs have a good spherical shape being the overall mean value around 1.08.


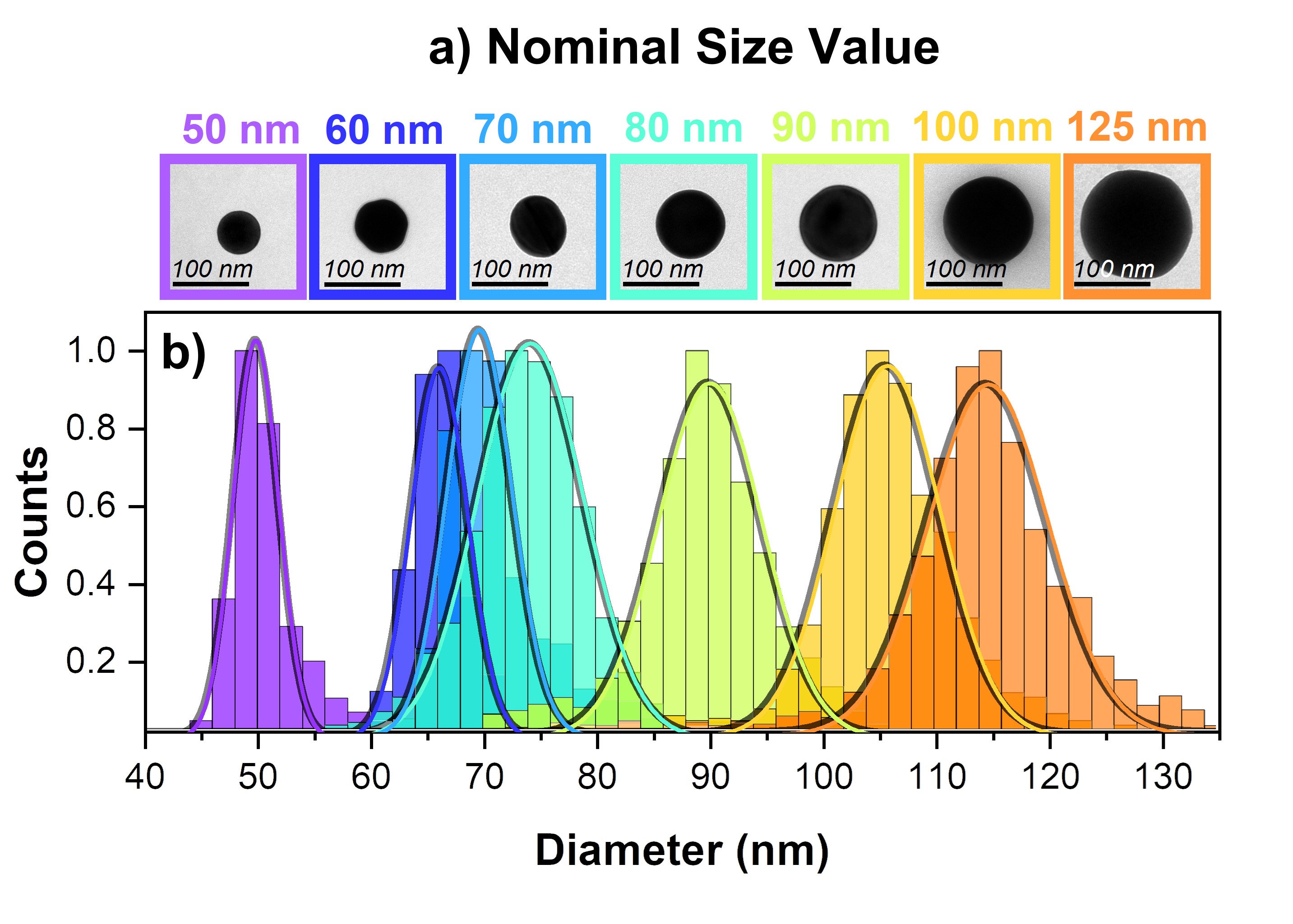


**Figure S3**: (a) Single nanoparticle TEM images of GNPs with different sizes; GNP diameters vary from 50 nm to 125 nm. As confirmed by the captured TEM images, all the nanoparticle lots studied present a very good roundness, about a 92%. (b) Histograms of the diameters of each nanoparticle lot; as outlined by the solid line for each nanoparticle lot, nanoparticles follow a Gaussian distribution with a standard deviation that is in good agreement with the nominal value given by the manufacturer.

|  | **TEM Size (nm)** | **Standard Dev. (nm)** |
| --- | --- | --- |
| **50 nm** | 49.7 | 0.6 |
| **60 nm** | 57.8 | 3.7 |
| **70 nm** | 69.5 | 0.7 |
| **80 nm** | 74.1 | 7.4 |
| **90 m** | 89.9 | 0.1 |
| **100 nm** | 103.3 | 3.3 |
| **125 nm** | 114.5 | 8.4 |

**Table S1**: TEM estimation of GNPs diameter obtained with CHT.

|  | **Aspect ratio** |
| --- | --- |
| **50 nm** | 1.07 |
| **60 nm** | 1.08 |
| **70 nm** | 1.15 |
| **80 nm** | 1.07 |
| **90 m** | 1.09 |
| **100 nm** | 1.1 |
| **125 nm** | 1.06 |

**Table S2**: TEM estimation of GNPs aspect ratio obtained based on CHT.

1. **Size Comparation between TEM and DF-SPS**

Table S3 gives the size values obtained for each batch of GNPs. The average difference between TEM and DF-SPS is 3 nm, a discrepancy of less than 3%.

|  | **TEM Size (nm)** | **DF-SPS Size (nm)** | **Discrepancy (nm)** | **Discrepancy (%)** |
| --- | --- | --- | --- | --- |
| **50 nm** | 49.7 | 49.5 | 0.2 | 0.4 |
| **60 nm** | 57.8 | 57.9 | -0.1 | 0.2 |
| **70 nm** | 69.5 | 68.2 | 1.3 | 1.9 |
| **80 nm** | 74.1 | 81.9 | -7.8 | 10 |
| **90 m** | 89.9 | 93.8 | -3.9 | 4.3 |
| **100 nm** | 103.3 | 101.6 | 1.7 | 1.6 |
| **125 nm** | 114.5 | 117.9 | -3.4 | 2.9 |

**Table S3**: Comparison between the particle diameters obtained with TEM and the ones obtained with DF-SPS

1. **Correlation between amplitude and wavelength of plasmonic GNPs**

The correlation between the amplitude and the position of the plasmonic peak for all nanoparticles sizes measured in this work is shown in Figure S4. As expected for spherical nanoparticles, we observed a positive linear relationship; this additional validation corroborates once again how the nanoparticles used in this work are high quality spherical nanoparticles.


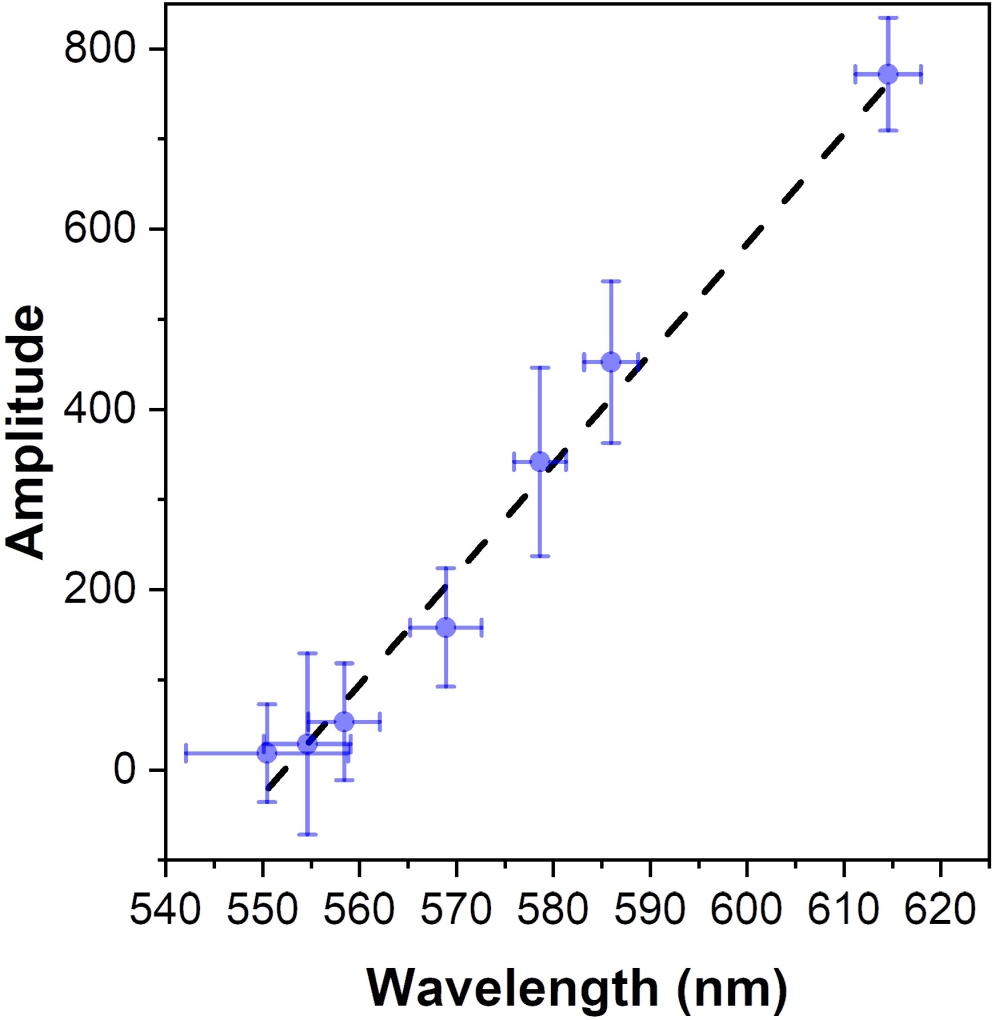


**Figure S4**: Correlation graph between amplitude normalized by the exposure time and the position of the plasmonic peak for each lot of nanoparticles. The blue line in the graph represents their linear relationship.

**References**

1. Rafael C. Gonzalez, Richard E. Woods & Steven L. Eddins. Digital Image Processing Using MATLAB, 3rd edition. (Gatesmark Publishing).

2. Bohren, C. F. & Huffman, D. R. Absorption and Scattering of Light by Small Particles. (Wiley, 1998). doi:10.1002/9783527618156.

3. Haiss, W., Thanh, N. T. K., Aveyard, J. & Fernig, D. G. Determination of size and concentration of gold nanoparticles from UV-Vis spectra. Anal Chem 79, 4215–4221 (2007).

4. Matzler, C. MATLAB functions for Mie scattering and absorption. IAP Res Rep 8, (2002).

5. Kreibig, U. & Fragstein, C. v. The limitation of electron mean free path in small silver particles. Zeitschrift für Physik 224, 307–323 (1969).

6. Johnson, P. B. & Christy, R. W. Optical Constants of the Noble Metals. Phys Rev B 6, 4370–4379 (1972).

7. Ku, H. H. Notes on the use of propagation of error formulas. Journal of Research of the National Bureau of Standards, Section C: Engineering and Instrumentation 70C, 263 (1966).

8. Yuen, H. K., Princen, J., Dlingworth, J. & Kittler, J. A Comparative Study of Hough Transform Methods for Circle Finding. in Procedings of the Alvey Vision Conference 1989 29.1-29.6 (Alvey Vision Club, 1989). doi:10.5244/C.3.29.
